# CMR findings in high endurance veteran athletes - a 247 subject study

**DOI:** 10.1186/1532-429X-18-S1-O38

**Published:** 2016-01-27

**Authors:** Viviana Maestrini, Ahmed Merghani, Stefania Rosmini, Andrew Cox, Heerajnarain Bulluck, Veronica Culotta, Mun Cheang, Marianna Fontana, Thomas A Treibel, Amna Abdel-Gadir, Sanjay Sharma, James Moon

**Affiliations:** 1 Cardiology, La Sapienza, Rome, Italy; 2 St George Hospital, London, UK; 3 Barts Heart Hospital, London, UK

## Background

Athletes demonstrate major adaptative changes in their hearts with high level of exercise. However the long term consequences of endurance training were not completely understood. We aimed to to explore the effect of long term endurance exercise on cardiac morphology and function, and diffuse fibrosis, and assess the significance of any discovered changes (correlation with documented ventricular arrhythmias).

## Methods

Veteran athletes (Vets) were >40, did competitive endurance exercise >10 years and had done multiple competitions. Controls were healthy volunteers (HV). Demographics, blood pressure, Bloods (hematocrit, renal function, lipids), rest ECG were collected. CMR at 1.5 T with T1 mapping (ShMOLLI and MOLLI) was performed using extracellular volume (ECV) quantification. The intracellular volume (1-ECV) and total cell volume (ICV × LVMass/1.05) were derived. Vets also had a 24-hour Holter. Scar was defined on LGE as "Overt" - in the compacted LV myocardium (ischaemic or non-ischemic pattern) and not limited to the RV insertion points or "minor" (RV scar, RV insertion point scar or papilliary muscle scar).

## Results

247 subjects were recruited: 158 Vet (71% male, mean ± SD age:55 ± 8 ys) and 89 HV (53% male, mean age 50 ± 13 ys). Vets (male and female) demonstrated increased LV volumes, mass and atria size, with no difference in ejection fraction.

**Figure 1:**
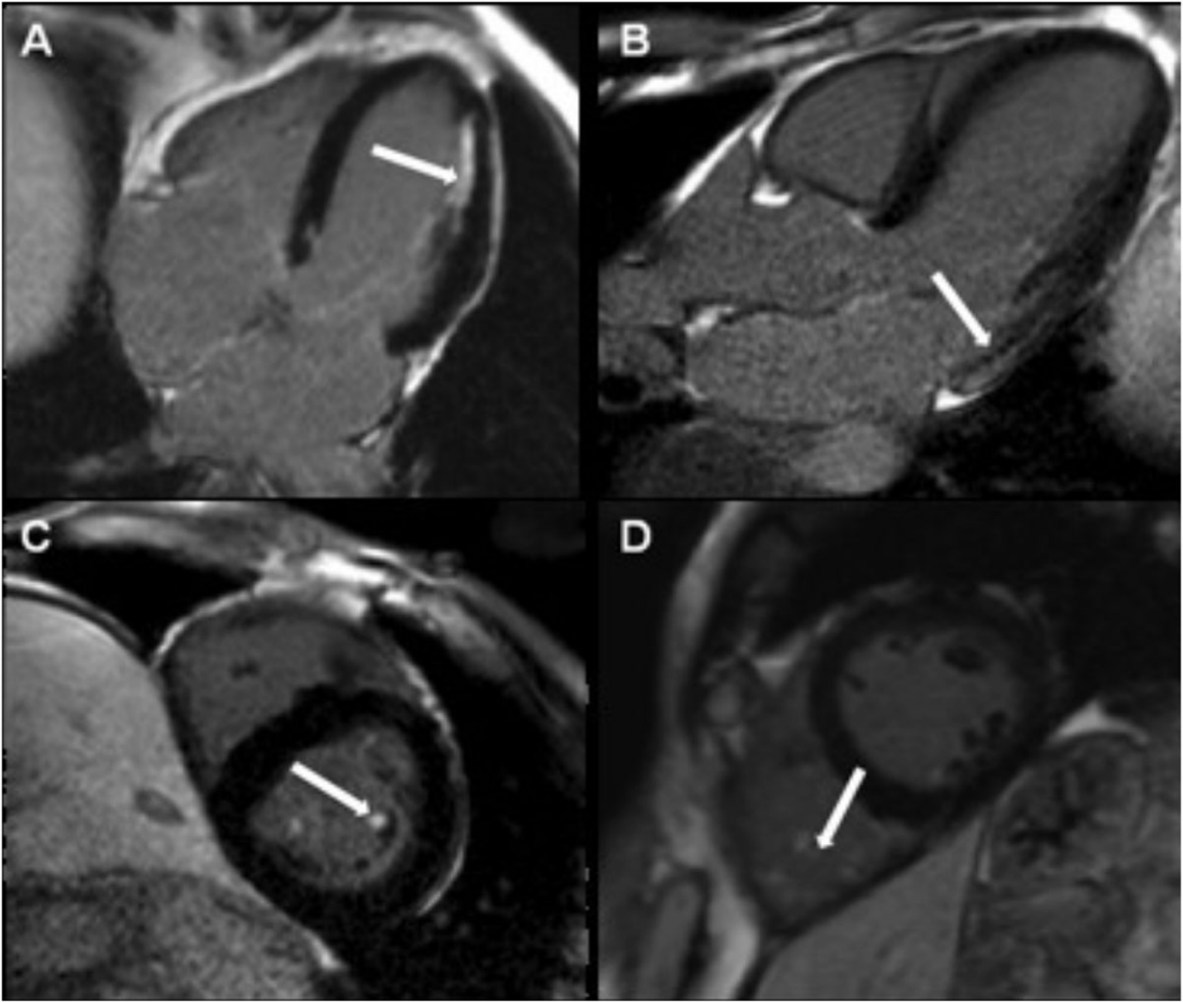
**Examples of different LGE pattern in veteran athletes: A) overt ischemic, B) overt non ischemic, c) minor, involving the papillary muscles or 4) minor, RV trabeculae**.

**LGE:** Healthy volunteers had no overt scar but 10% had minor scar - all of which was inferior RV insertion point. Vets had overt myocardial scar in 11.4%. This was 56% non-ischemic, 33% ischemic, 11% both. This was mainly males (15.2% vs 2.2%, p = 0.025). In addition, minor scar was high prevalence - inferior RV insertion point 19.6%, papillary muscles 27.8%, and right ventricle trabeculae 10.1% (Figure 1). These significant differences persisted when age and sex matched comparisons with HVs was made.

Overt LGE was significantly associated with non sustained ventricular tachycardia (OR = 5.4, 95% CI 1.2-24.9, p = 0.030).

**Figure 2:**
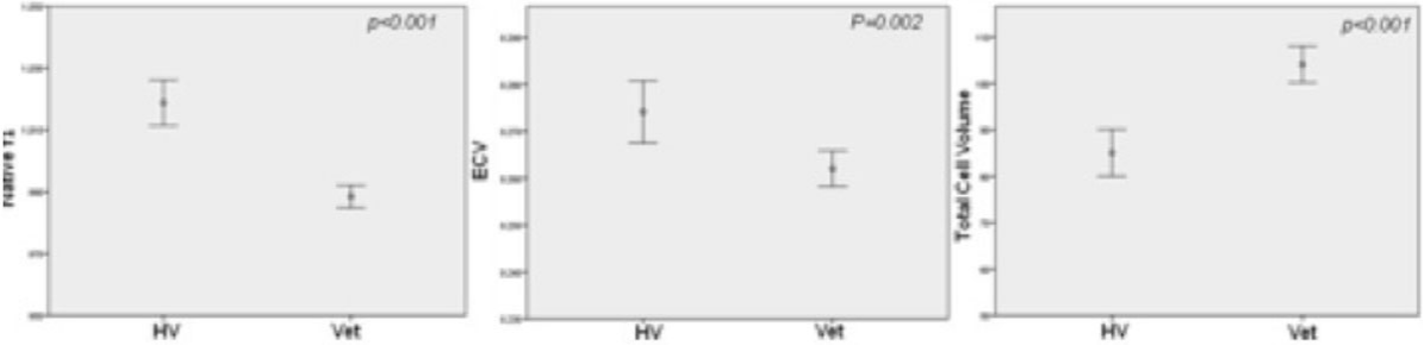
**Veteran athletes (Vet) has significantly lower native T1 (on the left), ECV (middle) and higher total cell volume (on the right) compared with healthy volunteers (HV)**.

Native T1 and ECV (drawn away from scar) were significantly **LOWER** in Vets. This was the same on MOLLI and ShMOLLI. MOLLI: native T1 988 ± 23 vs 1018 ± 36, p < 0.001; ECV 0.26 ± 0.024 vs 0.27 ± 0.03, p = 0.002; ShMOLLI: native T1 929 ± 26 vs 952 ± 29 ms, p < 0.001; ECV 0.26 ± 0.029 vs 0.28 ± 0.028, p < 0.00). The total cell volume were higher in Vets (104 ± 25 vs 85 ± 24 ml, p < 0.001), and specifically higher in the group with LV mass to volume ratio >1 (105 ± 22 vs 82 ± 23 ml, p = 0.002) (Figure 2). The total cell volume correlatee with MWT (r = 0.74 p < 0.001) and LA area (r = 0.48 p < 0.001).

## Conclusions

Veteran athletes have increased LV and atrial size and increased LV mass. The LVH in veterans has a lower ECV and T1 than health - suggesting hypertrophy (away from scar) is cellular rather than interstitial. Compared to age/sex matched controls, there is significant overt (11%), and minor (25%) scarring. Overt scar was highly associated with NSVT.

The impression therefore is that sustained athleticism causes scarring which is detrimental.

